# Energy Dispersion Induced Precisely Tunable Friction of Graphitic Interface

**DOI:** 10.1002/advs.202500378

**Published:** 2025-04-25

**Authors:** Zhao Liu, Hang Yang, Sen Wang, Jinxiong Wu, Wengen Ouyang, Junyan Zhang, Feng Luo

**Affiliations:** ^1^ State Key Laboratory of Solid Lubrication, Lanzhou Institute of Chemical Physics Chinese Academy of Sciences Lanzhou 730000 P. R. China; ^2^ School of Materials Science and Engineering Nankai University Tianjin 300350 P. R. China; ^3^ School of Civil Engineering Wuhan University Wuhan 430072 P. R. China; ^4^ State Key Laboratory of Water Resources Engineering and Management Wuhan University Wuhan 430072 P. R. China

**Keywords:** C‐EFM, electric coupling, energy dispersion, friction tuning, graphene

## Abstract

Precise control of friction at the nanoscale is crucial for developing efficient micro/nano‐electromechanical systems. This study presents a novel approach to manipulate friction in two‐dimensional materials using coupled direct current (DC) and alternating current (AC) electric fields. By applying a low‐amplitude AC bias atop a DC field, friction on monolayer graphene is continuously reduced without compensating the DC bias, while preserving the integrity of the graphitic interface. Theoretical analysis through the generalized Prandtl–Tomlinson model reveals a unique energy dispersion mechanism, where vertical resonance absorbs horizontal energy, minimizing sliding friction and enhancing interfacial durability. This approach addresses limitations in conventional electrically controlled friction methods, enabling precise device manipulation and offering new insights into frictional behavior and energy transmission.

## Graphical abstract

This study introduces a novel approach to precisely tune nanoscale friction on monolayer graphene using coupled DC/AC electric fields. Combining experiments (contact‐mode electrostatic force microscopy) and simulations (generalized Prandtl–Tomlinson model), vertical resonance is shown to disperse horizontal energy, reducing friction while preserving interface durability. The method overcomes limitations of single electric‐field control, enabling robust manipulation for advanced nanodevices.

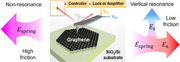

## 1 Introduction

As the demand for advanced devices with optimized functionalities grows, two‐dimensional (2D) layered materials have garnered widespread attention due to their exceptional physical properties.^[^ ^1^, ^2^, ^3^ ^]^ With the atomically flat yet structurally incommensurate surfaces, these materials exhibit low interfacial friction, making them ideal as natural solid lubricants.^[^ ^4^, ^5^ ^]^ The conception of “superlubricity,”^[^ ^6^ ^]^ characterized by nearly zero friction and wear, was first observed between two rotated graphite surfaces.^[^ ^7^, ^8^, ^9^ ^]^ It can be expanded to other layered 2D materials,^[^ ^10^ ^]^ graphite mesas,^[^ ^11^, ^12^, ^13^ ^]^ heterojunctions formed between graphene, h‐BN, MoS_2_, and metal surfaces,^[^ ^14^, ^15^, ^16^, ^17^, ^18^ ^]^ which also exhibit unique electronic properties such as unconventional superconductivity^[^ ^19^ ^]^ and twistronics.^[^ ^20^, ^21^ ^]^ These findings highlight a potential relationship between electronic and frictional interactions, suggesting the possibility of controlling friction through electric fields and overcoming the limitations of traditional parameter adjustments in enclosed interfaces.^[^ ^22^ ^]^ Early studies on electrically controlled friction began with silicon pn junctions under direct current (DC) electric fields.^[^ ^23^ ^]^ Later research expanded to 2D materials such as graphene,^[^ ^24^ ^]^ MoS_2_,^[^ ^25^ ^]^ MoSe_2_,^[^ ^26^ ^]^ h‐BN,^[^ ^27^ ^]^ and other heterostructures.^[^ ^28^ ^]^ However, the friction‐reduction effects of DC fields reach a plateau when the electrostatic force is fully compensated. Alternatively, alternating current (AC) electric fields have been explored,^[^ ^29^, ^30^ ^]^ though higher voltages often lead to surface degradation.^[^ ^13^, ^31^ ^]^ To address these challenges, this study investigates a novel approach combining DC and AC electric fields to enhance friction reduction through electric coupling.

**Figure 1:**
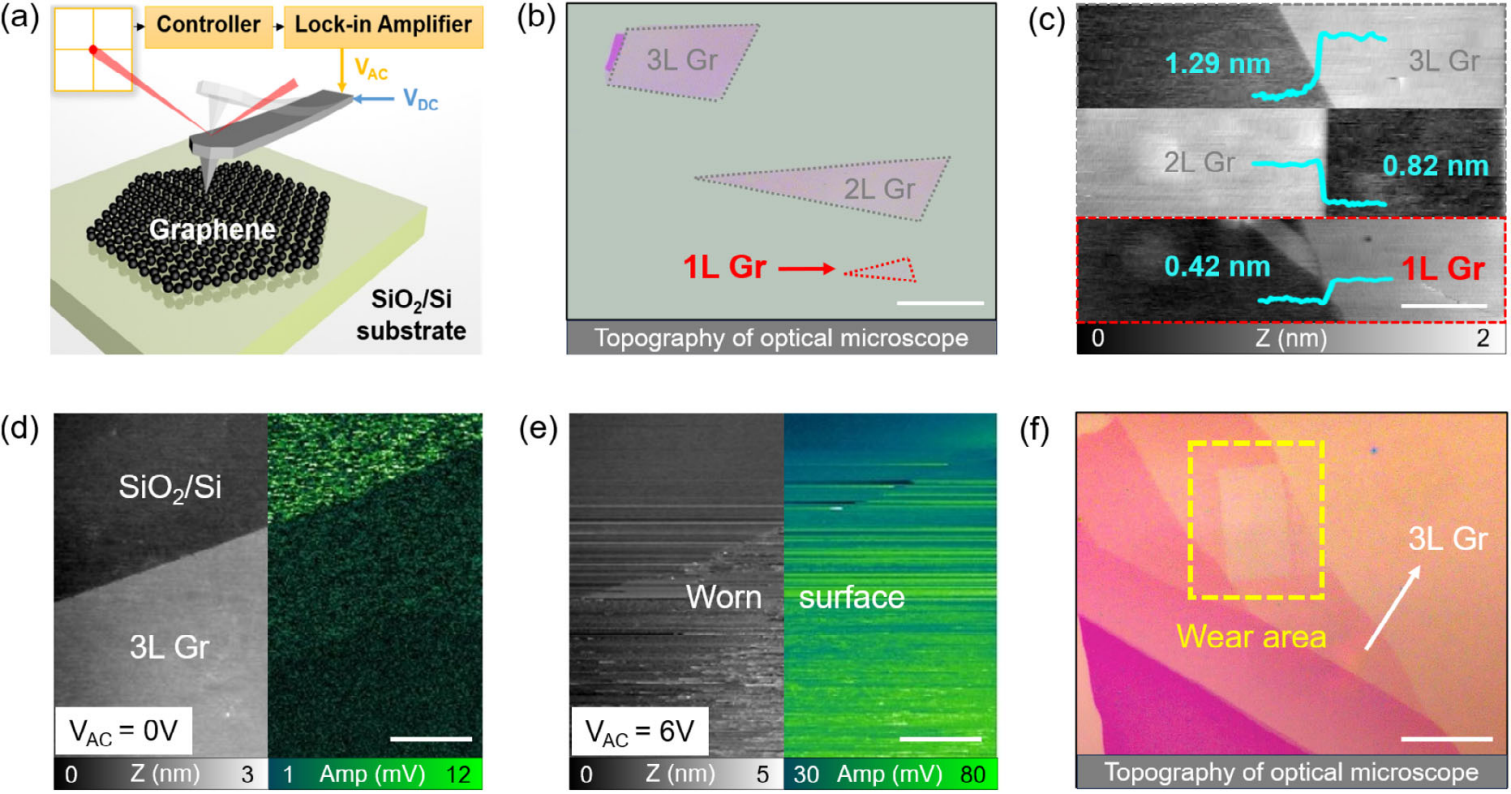
Schematic and characterization of graphene flakes on SiO_2_/Si. a) Experimental illustration under the coupled DC/AC electric fields by C‐EFM. b,c) Topography of 1‐3L graphene flakes observed under the optical microscope and AFM, respectively, with the line profiles of graphene steps in the inset of (c). d,e) Topography and amplitude of 3L graphene measured by C‐EFM under *V*_AC_ = 0 and 6 V, respectively (*F*_N_ = 10 nN, ω = 24 kHz, *V*_DC_ = 0 V). f) Zoomed‐out topography of worn graphene flakes observed under the optical microscope after the scanning of (e) at high AC bias. Scale bars for b): 20 µm, c): 1 µm, d,e): 4 µm, f): 30  µm.

In this study, the contact mode electrostatic force microscopy (C‐EFM) was used to simultaneously apply AC electric fields and DC biases,^[^ ^32^, ^33^ ^]^ as illustrated in **Figure** **1**a. The graphene with random layers were prepared by the mechanical exfoliation of natural graphite and transferred onto a 285 nm SiO_2_/Si substrate. We investigate the frictional manipulation induced by electric coupling in a monolayer graphene flake, with its location and height measurements measured by the optical microscope and an atomic force microscope (AFM) as shown in Figure 1b,c, respectively. The relationship between amplitude and friction was systematically investigated, with calibration details provided in Section S1 (Supporting Information). The generalized Prandtl‐Tomlinson model was employed to validate the observed inverse relationship between amplitude and friction. A special energy dispersion of vertical resonance is revealed with the tiding amplitude, leading to a continuous reduction in horizontal friction enhanced by the electric coupling effect. These findings provide critical insights into addressing frictional challenges in complex interfacial systems and enhancing nanoscale friction control.

## 2 Results and Discussion

### 2.1 Limitation of Friction Tuning Under a Single Electric Field

**Figure 2:**
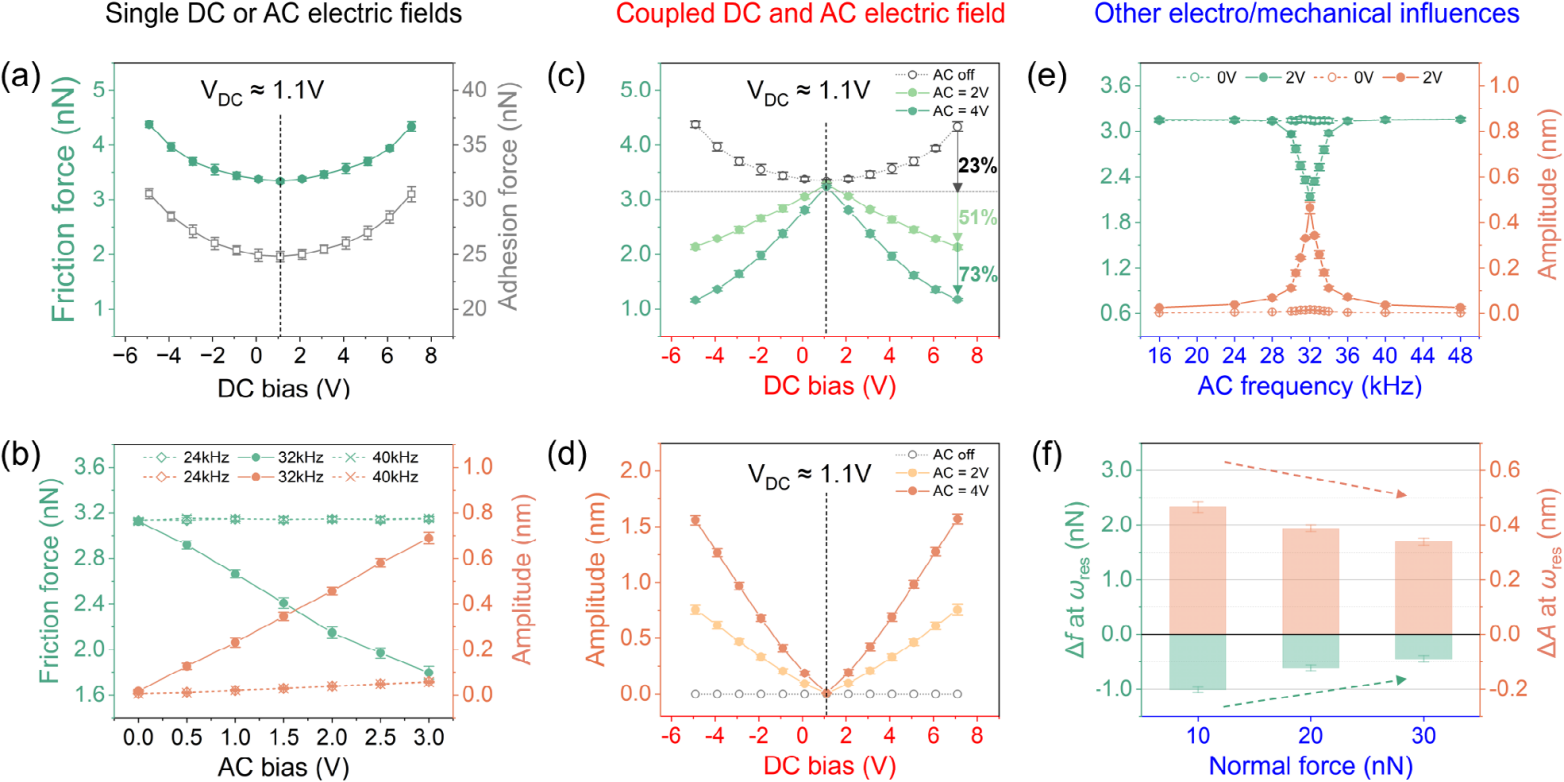
Tuning friction of monolayer graphene under different electric fields showing: a) The dependence of friction force (left y‐axis) and adhesion force (right y‐axis) on single DC bias with *F*_N_ = 10 nN. b) The dependence of friction force (left y‐axis) and amplitude (right y‐axis) on single AC bias under 32 kHz (solid symbols) and 24 kH/40 kHz (downward/upward open symbols) with *F*_N_ = 10 nN. c,d) The dependence of friction force and amplitude on coupled DC bias with AC bias fixed at 4 V, 2 V and off under ω = 30 kHz and *F*_N_ = 10 nN. e) The dependence of friction force (left y‐axis) and amplitude (right y‐axis) on AC frequency under *V*_AC_ = 0 V (open symbols) and *V*_AC_ = 2 V (solid symbols) with *F*_N_ = 10 nN, respectively. f) The dependence of friction force drop Δ*f* (left y‐axis) and amplitude increase Δ*A* (right y‐axis) on normal force at ω = 32 kHz and *V*_AC_ = 2 V.

The frictional response of monolayer graphene under single DC or AC electric fields was evaluated using C‐EFM in ambient conditions. We used the cantilever with the spring constant of *k* ≈ 0.2 N/m and the contact resonant frequency of ω_res_ ≈ 32 kHz (swept in Figure S2c, Supporting Information). The sliding velocity *v* and normal force *F*_N_ were kept constant at 1.6 µm/s and 10 nN, respectively. When a single DC bias is applied, the friction force followed a parabolic trend, reaching a minimum value at *V*_DC_ ≈ 1.1 V, directly affected by the electrostatic adhesion force (see **Figure** **2**a).^[^ ^25^, ^28^ ^]^ Beyond this compensated point, further reduction in friction was unattainable as the adhesion force was nearly offset, marking a clear limitation of DC control.

The effect of an AC bias on friction and amplitude was distinctly different, as shown in Figure 2b. When the AC frequency is away from *ω*_res_, the friction force and amplitude are almost unaffected by AC bias. However, at *ω*_res_, an increase in the AC bias leads to a linear decrease in the friction force and a linear increase in the amplitude. Notably, higher AC bias over 4 V were not considered to prevent sample surface damage, as excessive friction and wear at high voltages disrupt resonant contact stability.^[^ ^30^ ^]^ To test the durability against AC bias, we have applied *V*_AC_ from 0 to 6 V on trilayer graphene with fixed *V*_DC_ = 0 V and ω = 24 kHz (see details in Section S2 and Figure S1, Supporting Information). As shown in Figure 1d,e, even far away from ω_res_ accompanied with quite low normal force of 10 nN, the graphene flakes were suddenly worn after high *V*_AC_ sliding. The silicon substrate was exposed and obviously marked in the dashed box of Figure 1f. It can be speculated that the breakdown voltage for monolayer graphene will be no more than 6 V at the above AC conditions. These results highlight the challenges of single‐field control: DC biases are limited by fully compensated electrostatic forces, while AC biases risk sample degradation at elevated voltages.

### 2.2 Friction Force Manipulated by the Coupled DC/AC Effect

To overcome the limitations of single electric fields in manipulating friction on monolayer graphene, we applied a coupled DC and AC electric field. Measurements were conducted across a DC bias range of ‐5 to 7 V, where AC bias *V*_AC_ was fixed as 4 V, 2 V and off with ω = 30 kHz (see details for the resonant frequency setting in Section S3, Supporting Information), respectively. As shown in Figure 2c, activating the AC bias at 2 and 4 V results in a symmetrical linear friction force distribution with DC bias, peaking at *V*_DC_ ≈ 1.1 V. Compared to conditions without AC bias, the friction force at *V*_DC_ ≈ 7 V decreases by 50% under *V*_AC_ = 2 V and 75% under *V*_AC_ = 4 V, respectively. In contrast, the friction reduction dominated solely by DC bias is limited to 25% from *V*_DC_ ≈ 7 V to 1.1 V. These results demonstrate that neither single DC (e.g., *V*_AC_ = 0 V) nor AC bias alone (e.g., *V*_DC_ = *V*_CPD_) is sufficient for effective friction manipulation.

Simultaneously, the amplitude measurements were taken using C‐EFM as shown in Figure 2d. It exhibits a symmetrical linearity with DC bias, reaching a minimum at *V*_DC_ ≈ 1.1 V. According to the principle of the Kelvin probe force microscopy (KPFM), nearly zero amplitude corresponds to the compensated potential difference in the system, measured at –1.06 V between the Pt/Ir‐coated tip and the graphene surface (the measurement details are presented in Section S4 and Figure S3, Supporting Information). The amplitude slope with DC bias steepened from *V*_AC_ = 2 to 4 V, exhibiting an opposite trend to the friction force. By utilizing a coupled DC and AC bias, we achieve significant friction reduction beyond the electrostatic limitations of single‐field methods, while preserving the integrity of the graphene surface. This approach addresses the challenges faced by single DC or AC control methods, offering a more robust and precise strategy for friction manipulation.

### 2.3 The Negatively Amplitude‐Induced Friction Force

To further attempt the manipulation of the friction force through the electric coupling, it is worth examining the amplitude of the activated cantilever based on the negatively correlated amplitude‐frictional behaviors observed in Figure 2c,d. The resonant principle of C‐EFM is similar to that of the piezo‐response force microscopy (PFM), where the electrostatic force *F*_el_ can be separated from chemical or van der Waals forces by modulating an AC electric field to oscillate the cantilever, organized as:^[^ ^34^ ^]^

(1) $$\begin{array}{c} \begin{array}{cc} F_{\text{el}}= & -\frac{1}{2}\frac{dC}{dz}\left(\Delta V_{\text{DC}}^{2}+\frac{1}{2}V_{\text{AC}}^{2}\right)-\frac{dC}{dz}\Delta V_{\text{DC}}V_{\text{AC}}\sin\omega t\\ & +\frac{1}{4}\frac{dC}{dz}V_{\text{AC}}^{2}\cos2\omega t \end{array} \end{array}$$

where d*C*/d*z* is the capacitance gradient, Δ*V*_DC_ is the real‐time contact potential equal to the system's compensated potential minus the tip‐sample potential difference (i.e., *V*_DC_ − *V*_CPD_), *t* is time. The first part in Equation (1) represents the DC component, which is proportional to $\Delta V_{\text{DC}}^{2}$ and acts as the electrostatic adhesion when AC bias off. The second term, proportional to Δ*V*_DC_ when *V*_AC_ is fixed, is AC_*ω* term and generates the resonant amplitude. The last term, higher‐order AC_2ω, can be negligible in this context. Evidently, AC_ω term demonstrates the enhanced amplitude effect due to the interplay of DC and AC biases, which coincides with friction reduction. This interplay underscores the symbiotic relationship between amplitude and friction in this system.

Furthermore, the negatively correlated friction‐amplitude behaviors can be further tuned by varying parameters. As shown in Figure 2e, altering AC frequency from 16 to 48 kHz affects the friction force. At *V*_AC_ = 2 V, an extreme point for both friction force and amplitude occurs at ω_res_, but for minimum and maximum, respectively. Outside the ω_res_ ± 4 kHz range, the friction force returns to 3.1 nN, as stable as the non‐resonant value of monolayer graphene.^[^ ^30^, ^35^ ^]^ Similarly, the amplitude varies inversely with friction as frequency deviates from ω_res_. Both friction and amplitude exhibit minimal dependence on AC frequency, highlighting the need for appropriate AC frequency ranges (e.g., ω_res_ ± 4 kHz) and sufficient AC bias values (e.g., *V*_AC_ > 0 V).

Additionally, the normal force *F*_N_ also plays a significant role in influencing friction and amplitude. In Figure 2f, larger normal force weakens the friction drop Δ*f* at ω_res_ with *V*_AC_ = 2 V, which is defined as the friction difference between *V*_AC_ = 0 V and 2 V (see further details in Section 5 and Figure S4, Supporting Information). Similarly, the increase in amplitude Δ*A* diminishes under higher loading due to enhanced forced vibration. Thus, applying greater normal force alters both friction and amplitude while maintaining their inverse relationship, underscoring the critical balance of parameters required for precise tuning in this coupled DC/AC system.

### 2.4 Dynamic Adhesion Energy Obtained from DMT Fit

**Figure 3:**
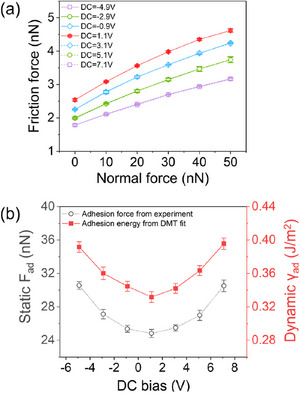
DMT fit of friction force under coupled DC/AC bias (*V*_AC_ = 2 V, ω = 30 kHz): a) Normal‐friction force curves of monolayer graphene tuned by DC bias. b) Adhesion force *F*_ad_ and energy γ_ad_ obtained from the experiment and DMT fit with DC bias, respectively.

Adhesion force plays a critical role in friction, particularly at the nanoscale, where it becomes a dominant surface interaction.^[^ ^36^, ^37^ ^]^ As previously discussed, the electronic friction influenced by DC bias is primarily linked to the electrostatic component of adhesion force *F*_el_.^[^ ^25^, ^38^ ^]^ However, the effect of adhesion force on friction behaviors under AC electric field is lack of comprehensive investigations.^[^ ^29^, ^39^ ^]^ The main challenge arises from the limitations of force spectroscopy in capturing static adhesion forces during dynamic oscillatory scanning. To address this, adhesion energy can be determined using the Derjaguin–Muller–Toporov (DMT) contact mechanics model as Equation (2):^[^ ^28^, ^40^ ^]^

(2) $$\begin{array}{c} \begin{array}{cc} f & =\pi {\left(\frac{3R}{4}\right)}^{\frac{2}{3}}\cdot \frac{\tau }{E_{\text{eff}}^{\frac{2}{3}}}\cdot {\left(F_{N}+2\pi R\gamma \right)}^{\frac{2}{3}}\\ & =\mu_{\text{DMT}}\cdot {\left(F_{N}+2\pi R\gamma \right)}^{\frac{2}{3}} \end{array} \end{array}$$

where *f* and *F*_N_ are the friction force and normal force, respectively; *R* is the tip radius; *τ* is the shear stress; *E*_eff_ is the effective modulus of the combined tip‐sample system; γ is the adhesion energy. Since the shear components including τ is not focused here, we define a normalized shear stress µ_DMT_ to merge the variables together before (*F*_N_ + 2πRγ) for simplicity. Thus, the dynamic adhesion energy γ_ad_ under the coupled DC/AC electric field can be acquired by the DMT fitting formula. The friction‐normal force curves under the different DC bias are first measured as shown in **Figure** **3**a, with the normal force range of 0 ∼ 50 nN, where the AC bias and AC frequency are set as 2 V and 30 kHz, respectively. Afterwards, the curves will be analyzed through the DMT fit function to extract the adhesion energy from Equation (2).

With the approximate tip radius of ∼15 nm from Figure S2 (Supporting Information), the dynamic adhesion energy γ_ad_ is obtained under DMT fit and shown in Figure 3b above. The fitted adhesion energy with DC bias exhibits a trend between parabolic and “V‐shaped.” This indicates that adhesion remains sensitive to coupled DC/AC electric fields, which activate resonance amplitude as described in Equation (1). However, the adhesion force does not strongly influence friction as the positive correlation under the static adhesion force *F*_ad_ (see Figure 2a). Interestingly, while the electrostatic component of dynamic adhesion force sustains the resonant amplitude, it no longer dominates friction behavior, leaving the precise mechanism of friction reduction under electric‐coupling conditions an open question.

### 2.5 Simulated Amplitude and Friction Established by PT Model

**Figure 4:**
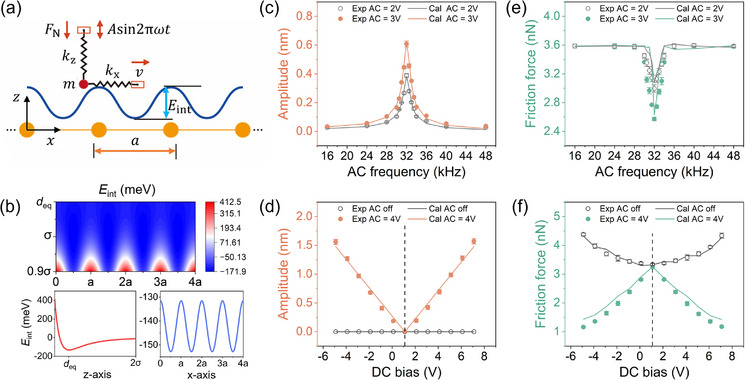
Numerical simulation of amplitude and friction. a) Illustration of the generalized PT model. b) The calculated *E*_int_ map, with the extracted variations along z‐axis (*x* = *a*) and x‐axis (*z* = *d*_eq_) shown in below, respectively. c,d) Calculated and experimental amplitudes with ω and *V*_DC_, respectively. e,f) Calculated and experimental friction forces with *ω* and *V*_DC_, respectively. [Correction added on 29 April 2025, after first online publication: figure 4 image is updated in this version.]

To complement the experimental results, we conducted numerical simulations using a generalized 2D Prandtl–Tomlinson (PT) model as illustrated in **Figure** **4**a (see further details in simulation methods). A particle with mass *m* is dragged by a spring moving at a constant velocity *v* in the x‐direction, and periodically vibrated by another spring in the z‐direction, with the respective spring constants denoting as *k*_x_ and *k*_z_. The choice of the potential energy surface is crucial for accurately simulating the frictional force. Previous research has established a suitable potential energy surface *E*_int_ to describe the frictional behavior of layered materials, which effectively mimics the results of 3D system^[^ ^41^ ^]^ (see further details in simulation methods). In Figure4b, *E*_int_ periodically fluctuates with the lattice constant *a* and reaches the minimum value at the equilibrium position *d*_eq_. Combining with the elastic potential energy *E*_x_ and *E*_z_ for both springs, the total potential energy *E*_tot_ of the system can be expressed as:

(3) $$\begin{array}{ccc} E_{\text{tot}}\left(x,z,t\right) & = & E_{\text{int}}\left(x,z\right)+E_{x}+E_{z}\\ & = & E_{\text{int}}\left(x,z\right)+\frac{1}{2}k_{x}(\mathrm{vt}-x)2+\frac{1}{2}k_{z}{\left(A\sin2\pi \omega t-z\right)}^{2} \end{array}$$

where *k*_x_ and *k*_z_ represent the corresponding spring stiffness (see **Figure** **4**a), and (*x*, *z*, *t*) denotes the particle's coordinates and time. By referring to the simple linear forced vibration and considering the external excitation as a bilinear polynomial of DC and AC bias, we can derive theformula for the amplitude *A* in terms of the coupled electric field:

(4) $$\begin{array}{c} \begin{array}{c} A=\frac{\alpha \left|V_{\text{DC}}-V_{\text{CPD}}\right|\cdot V_{\text{AC}}}{\sqrt{{\left(1-\frac{\omega^{2}}{\omega_{\text{res}}^{2}}\right)}^{2}+{\left(\frac{2\zeta \omega }{\omega_{\text{res}}}\right)}^{2}}} \end{array} \end{array}$$

where *α* is a linea coefficient to be fitted, *V*_CPD_ is set as 1.1 V from KPFM measurements, and *ζ* is a damping coefficient smaller than 1. The formula parameters are fitted using the amplitude‐frequency‐voltage relationship under *V*_AC_ = 2 V in Figure 4c, and the data for *V*_AC_ = 3 V is predicted using Equation (4). By selecting appropriate *α*, ω_res_ and *ζ* (the selection of simulation parameters see details in Section S6 and Figure S5, Supporting Information), Equation (4) can accurately describe how single AC frequency or bias, and the coupled electric field affect the amplitude (see Figure 4c and 4d). The close agreement between the formula and experiments suggests that the system is indeed undergoing the linear forced vibration.

Subsequently, the friction forces under different electric fields are simulated using the generalized PT model. The motion of the particle in Figure 4a is determined by the modified Langevin equation as Equations (5) and (6):^[^ ^42^ ^]^

(5) $$\begin{array}{c} \begin{array}{c} m\frac{d^{2}x}{dt^{2}}+m\mu_{x}\left(\frac{dx}{dt}-v\right)+\frac{\partial E_{\text{tot}}\left(x,z,t\right)}{\partial x}=\xi_{x}\left(t\right) \end{array} \end{array}$$

(6) $$\begin{array}{c} \begin{array}{c} \begin{array}{ccc} m\frac{d^{2}z}{dt^{2}}+m\mu_{z}\left(\frac{dz}{dt}-2\pi \omega A\cos2\pi \omega t\right) & +\frac{\partial E_{\text{tot}}\left(x,z,t\right)}{\partial z} & =-F_{N}+\xi_{z}\left(t\right) \end{array} \end{array} \end{array}$$

where *µ*_x_ and *µ*_z_ are damping coefficients, and *ξ*_x_(*t*) and *ξ*_z_(*t*) are Gaussian distributed random noise satisfying the fluctuation‐dissipation theorem (See the Simulation and calculation Method for details). A fourth‐order Runge–Kutta algorithm is used to numerically solve the Langevin equation at 300 K.^[^ ^43^ ^]^ Compared with Ermak's algorithm, it effectively captures stochastic processes when implemented with an appropriately small‐time step, offering enhanced accuracy, robustness and suitability for our study. Afterwards, the mean value of the lateral force over multiple periods 〈*F*_L_〉,^[^ ^44^ ^]^ i.e., the friction force *f*, is calculated as:

(7) $$\begin{array}{c} f=\langle F_{L}\rangle =\frac{1}{T}\int_{0}^{T}k_{x}\left(vt-x\right)dt \end{array}$$

where T is a period of the particle's steady sliding. When only AC bias is applied, the electric field in this model directly affects the friction force through the amplitude. As shown in Figure 4e, the friction force exhibits a steep decline as ω approaches resonance. Under different *V*_AC_, the friction force significantly decreases within almost the same frequency range (28∼36 kHz), in comparison to the amplitude surge (see Figure 4c). The simulation results show a resonance friction force that negatively correlates with the amplitude, consistent with the experimental findings and previous theoretical studies.^[^ ^31^, ^43^, ^45^ ^]^

To describe the strong correlation between adhesion force and DC bias, the key parameter *ϵ* in the expression of *E*_int_ is assumed to be a quadratic function of *V*_DC_ in this model (See Equations (8) and (9) in the simulation methods). The simulation results support the approximate quadratic relationship between frictional force and DC bias observed in the static compensation regions (see Figure 4f). Considering the combined effects of *V*_DC_ on *ϵ* and the influence of the coupled DC and AC bias on the amplitude, the simulation results show a trend opposite to the positive correlation observed with DC bias alone. The influence of AC bias on the amplitude is amplified by DC bias, resulting in a stronger resonance amplitude that counteracts the effect of DC electrostatic adhesion. This leads to a greater decrease in friction force compared to applying AC voltage alone.

### 2.6 Coupled Energy Dispersion from Laterally to Vertically

**Figure 5:**
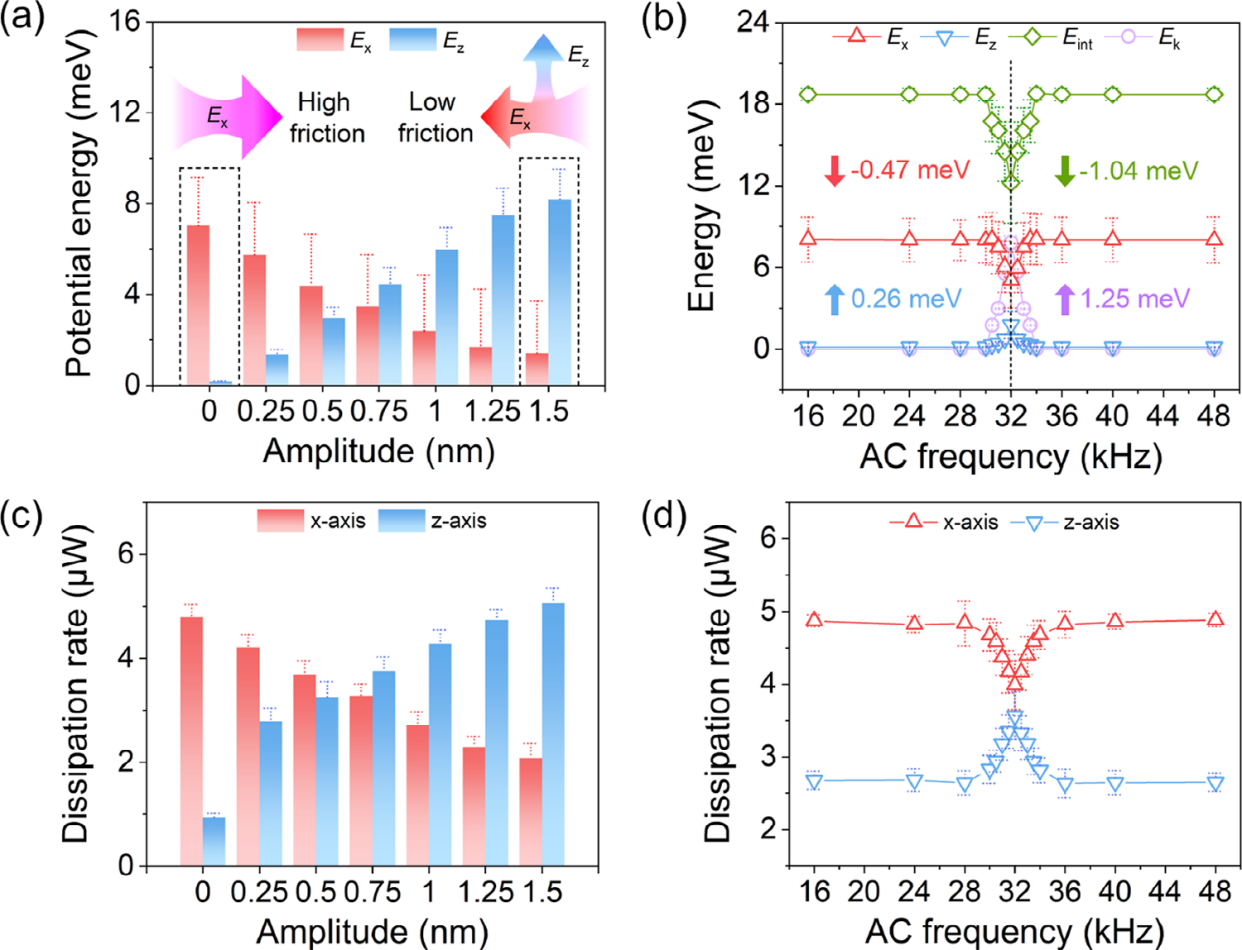
Coupled horizontal and vertical energy dissipation calculated by the generalized PT model. a,,b) The dependence of the springs' potential energy *E*_x_ and *E*_z_ on amplitude (ω = 30 kHz, *V*_AC_ = 4 V, *V*_DC_ = 1.1 ∼ 7.1 V (Δ*V*_DC_ = 1 V)) and AC frequency (*V*_AC_ = 2 V, *V*_DC_ = 0 V, ω = 16 ∼ 48 kHz), respectively. c,d) The dependence of the potential energy dissipation rate *P*_x_ and *P*_z_ on amplitude and AC frequency (same parameters with (a) and (b)), respectively.

To further elucidate the energy dissipation process during the electric coupling, we calculated the springs' potential energy *E*_x_ and *E*_z_ from Equation (3). Typically, both *E*_x_ and *E*_z_ should be mutually independent due to the orthogonal displacement vectors. However, owing to the modified Langevin equations of Equations (5) and (6), the springs' movements along x and z directions reveal a coupling with each other. When increasing the amplitude as shown in **Figure** **5**a, the horizontal *E*_x_ is suppressed, with the excess energy dissipated vertically as *E*_z_. Calculations in Figure 5b displays the simultaneously fading *E*_x_ and growing *E*_z_ when approaching the resonant AC frequency, directly aligning with the negatively correlated friction and amplitude behaviors in Figure 2e. Notably, the decrease in horizontal *E*_x_ (–0.47 meV) is not completely equivalent with the increase in vertical *E*_z_ (0.26 meV) at ω_res_. Indeed, the elastic potential energy *E*_x_ and *E*_z_ stored in the spring is not the whole energy of this system (spring plus particle). The extra decrease in *E*_x_ translates into a significant increase in the kinetic energy $E_{k}=1/2m\left(v_{x}^{2}+v_{z}^{2}\right)$ of the particle as it approaches resonance, where *v*_x_ and *E*_z_ refer to the speed of the particle in x and z direction, respectively. Even the interlayer potential energy *E*_int_ is partially converted into *E*_k_. Considering the energy for both particle and spring (see Figure 5b), the decrease of *E*_x_ and *E*_int_ is balanced by the increase of *E*_z_ and *E*_k_, resulting in a total energy exchange of 1.51 meV at ω_res_.

Building on this energy analysis, we also extracted the energy dissipation rates of the spring system *P*, along the x‐ and z‐axes using the generalized PT model. These rates exhibit similar variations with increasing amplitude as that of potential energy (see Figure 5c), specifically a decreasing Δ*P*_x_ accompanied by an increasing Δ*P*_z_. Comparing the drop of Δ*P*_x_ = –0.886 µW and the increase of Δ*P*_z_ = 0.889 µW in average at ω_res_ from Figure 5d, we observe an almost equivalent energy exchange between the vertical *E*_x_ and horizontal *E*_z_. This balance highlights the energy transmission mechanism underlying negatively correlated friction‐amplitude behavior: vertical resonance absorbs dissipation energy, reducing horizontal sliding energy and enhancing interfacial durability (as illustrated in the inset of Figure 5a). For example, phonons generated during nonlinear sliding can, in turn, induce resonant tip vibrations and jumping friction forces at specific velocities.^[^ ^46^ ^]^ Instead, our results suggest that by precisely controlling energy transmission channels through electric coupling effect — akin to directing traffic through specific lanes — enables efficient energy dispersion at sliding interfaces, facilitating robust and tunable friction control.

## 3 Conclusion

Our study presents a novel method for precisely tuning the friction force of monolayer graphene through a coupled DC/AC electric field. This approach enhances the resonant effect while mitigating adhesion influences, addressing the limitations of friction control using single DC or AC biases. The coupled DC/AC electric field induces a negatively correlated friction‐amplitude relationship, enabling flexible friction adjustments by modulating AC parameters and normal force, with corresponding variations in amplitude. Simulations based on the generalized PT model validate that friction reduction is linked to energy dispersion from horizontal to vertical directions under resonant conditions. This electric coupling effect not only offers precise control over the friction of micro‐ and nanoscale devices for advanced lubricative technologies, but also introduces a novel framework for understanding energy transmission mechanisms, paving the way for robustly tunable friction systems.

## 4 Experimental Section

### Sample Preparation

The graphene with random layers were prepared by the mechanical exfoliation of natural graphite and transferred onto a 285 nm SiO_2_/Si substrate. An optical microscope was used to roughly locate the graphene flakes,^[^ ^47^ ^]^ while the layer number was further identified by an atomic force microscope (AFM).

### Experimental

The main experiments were carried out with an commercial AFM equipment (Bruker, Dimension Icon) in ambient environment. The friction forces were measured via a soft and conductive probe (Bruker, SCM‐PIC, with the Pt/Ir coated tip, the spring constant *k* ≈ 0.2 N/m, the contact resonance frequency ω_res_ ≈ 32 kHz, swept in Figure S2, Supporting Information). Both amplitude and friction force were simultaneously obtained through the contact mode electrostatic force microscopy (C‐EFM). An AC bias and frequency was applied on the tip to reach the resonant effect, with the setting range of 0∼4 V and 16∼48 kHz, respectively. The graphene sample was scanned at a speed of 1.6 µm/s at the normal forces *F*_N_ of 10 nN. Furthermore, an additional DC bias was also applied on the tip with the range of –4.9∼7.1 V, depending on the shift of the contact potential difference toward 1.1 V, which was measured by Kelvin probe force microscopy (KPFM, as shown in Figure S3, Supporting Information) through the tapping mode and a hard probe (Bruker, SCM‐PIT, the spring constant *k* ≈ 2.8 N/m, the free resonance frequency ω_0_ ≈ 75 kHz). Additionally, we measured the adhesion force from the maximum value of the force‐distance curve during the force spectroscopy, while the adhesion energy was calculated by DMT fit function with the data from the corresponding friction‐normal force curves.

### Simulation

In Equation (3), *E*_int_ can be written as:^[^ ^41^ ^]^

(8) $$\begin{array}{c} E_{\text{int}}\left(x,z\right)=E_{0}\left(z\right)+\sum_{n=1}^{\infty }E_{n}\left(z\right)\cos\frac{2\pi n}{a}x \end{array}$$

where *a* is the period of substrate showing in Figure 4a, (*x*, *z*) is the particle position, *E*_0_(*z*) and *E*_n_(*z*) are expanded as:

(9) $$\begin{array}{c} E_{0}\left(z\right)=\pi \varepsilon \frac{\sigma }{a}\left[\frac{63}{64}{\left(\frac{\sigma }{z}\right)}^{11}-\frac{3}{2}{\left(\frac{\sigma }{z}\right)}^{5}\right] \end{array}$$

(10) $$\begin{array}{c} E_{n}\left(z\right)=2\varepsilon \sum_{n=1}^{\infty }\frac{1}{n}e^{-\chi }\left[{\left(\frac{\chi }{z}\sigma \right)}^{12}\phi_{6}\left(\chi \right)-{\left(\frac{\chi }{z}\sigma \right)}^{6}\phi_{3}\left(\chi \right)\right] \end{array}$$

where *σ* and *ϵ* = *ϵ*_0_ + *λ*(*V*_DC_ − 1.1)^2^ are the respective equilibrium distance and strength parameters of the Lennard‐Jones (LJ) interactions. Considering the LJ potential is commonly applied to simulate dynamic atomic‐scale interactions on a 2D surface, yet it is computationally expensive for large‐scale tribological simulations at super‐low sliding velocities in our experimental conditions. To overcome this limitation, we extended the PT model by incorporating both lateral and vertical interactions between the tip and the surface into the potential energy term.^[^ ^48^ ^]^ Then, the specific form of the potential *E*_0_(*z*) in Equations (9), derived from the LJ potential, produces results equivalent to those of the standard LJ potential while significantly reducing computational cost. This analytical approach enables us to perform simulations under conditions comparable to experimental observations and to develop a generalized PT model for broader applications. Additionally, ϕ_6_(χ), ϕ_3_(χ) and χ are expanded as:

(11) $$\begin{array}{c} \phi_{6}\left(\chi \right)=\frac{1}{3840}\left[\frac{945}{\chi^{11}}+\frac{945}{\chi^{10}}+\frac{420}{\chi^{9}}+\frac{105}{\chi^{8}}+\frac{15}{\chi^{7}}+\frac{1}{\chi^{6}}\right] \end{array}$$

(12) $$\begin{array}{c} \phi_{3}\left(\chi \right)=\frac{1}{8}\left[\frac{3}{\chi^{5}}+\frac{3}{\chi^{4}}+\frac{1}{\chi^{3}}\right] \end{array}$$

(13) $$\begin{array}{c} \chi =\frac{2\pi n}{a}z \end{array}$$

Under linear forced vibration, the relationship between the system's amplitude *A* and external amplitude *A*_0_ can be written as:

(14) $$\begin{array}{c} A=\frac{A_{0}}{\sqrt{{\left(1-\frac{\omega^{2}}{\omega_{\text{res}}^{2}}\right)}^{2}+{\left(\frac{2\zeta \omega }{\omega_{\text{res}}}\right)}^{2}}} \end{array}$$

(15) $$\begin{array}{c} A_{0}=\alpha \left|V_{\text{DC}}-V_{\text{CPD}}\right|\cdot V_{\text{AC}} \end{array}$$

Here, ω_res_ is the contact resonance frequency of the system, and ζ is the damping ratio. Considering the external amplitude *A*_0_ obtained as linear functions of both DC and AC bias, and multiplying them together while taking into account that there is no amplitude when AC off. Then, just simply multiply Equations (14) and (15) to obtain Equation (4).

For the parameter values, we took *a* = 0.246 nm, *σ* = 0.247 nm, *ϵ*_0_ = 7.6 eV, *λ* = 2.25, *v*_x_ = 1.6 µm/s, *k*_x_ = 37.74 N/m, *k*_z_ = 0.073 N/m, *α* = 0.0079 nm/V^2^, *ω*_res_ = 32 kHz, *ζ* = 0.0189, *m* = 7.1289 × 10^−11^ kg, µ_x_ = 1.5228$\sqrt{k_{x}/m}$, µ_z_ = 1.0931$\sqrt{k_{z}/m}$, *T* = 300 K.

## Conflict of Interest

The authors declare no conflict of interest.

## Author Contributions

Z.L. and H.Y. are the co‐first authors. Z.L. conceived the original idea and the experiments. W.O. proposed the theoretical model; H.Y. and S.W. conducted the numerical simulations; Z.L., H.Y. and W.O. wrote the manuscript; J.X.W. provided the samples; F.L. and J.Y.Z acquired the research funding. All authors discussed the results and revised the manuscript.

## Supporting information

Supporting Information

## Data Availability

The data that support the findings of this study are available from the corresponding author upon reasonable request.
