# Supplementary material for: Energy Dispersion Induced Precisely Tunable Friction of Graphitic Interface
Source: Adv Sci (Weinh). 2025 Apr 25;12(23):2500378. doi: 10.1002/advs.202500378 (PMC12199516; doi:10.1002/advs.202500378)
Supplement: Supplementary file 1 — Supporting Information [file ADVS-12-2500378-s001.pdf]

ADVANCED SCIENCE
Open Access

Supporting Information

for *Adv. Sci.*, DOI 10.1002/advs.202500378

Energy Dispersion Induced Precisely Tunable Friction of Graphitic Interface

Zhao Liu, Hang Yang, Sen Wang, Jinxiong Wu, Wengen Ouyang*, Junyan Zhang and Feng Luo**

Supporting information for: Energy dispersion induced precisely tunable friction of graphitic interface

Zhao Liu,*[a],[b] Hang Yang,[c] Sen Wang,[c] Jinxiong Wu,[b] Wengen Ouyang*,[c] Junyan Zhang,[b] Feng Luo,*[b]

This Supporting Information document includes the following sections:

1. Calibration of force and amplitude in C-EFM
2. The graphene failure after higher AC bias scanning
3. Cantilever characterization and contact resonant frequency sweep
4. Work function of monolayer graphene measured by KPFM
5. Full data of normal force tuning on friction via electric coupling
6. The selection of fitted simulation parameters

[a] Dr. Zhao Liu*, Prof. Junyan Zhang
State Key Laboratory of Solid Lubrication
Lanzhou Institute of Chemical Physics
Chinese Academy of Sciences
Lanzhou 730000, P.R. China
E-mail: zhaoliu@licp.cas.cn

[b] Dr. Zhao Liu, Prof. Jinxiong Wu, Prof. Feng Luo*
School of Materials Science and Engineering
Nankai University
300350 Tianjin, P. R. China
E-mail: feng.luo@nankai.edu.cn

[c] Hang Yang, Sen Wang, Prof. Wengen Ouyang*
School of Civil Engineering
Wuhan University
430072 Wuhan, P. R. China
E-mail: w.g.ouyang@whu.edu.cn

1. Calibration of force and amplitude in C-EFM

The amplitude we measured is stemmed from the electrostatic adhesion force via C-EFM. If we put two voltages as V_{AC} and V_{DC} with the amplitude in voltage together, it may decrease the readability of this paper. Anyway, it is necessary to provide a clear explanation for this issue. The formula we transfer the measured amplitude of voltage into distance is shown as:

$$A_{distance} = A_{voltage} \times S_{\mathrm{N}} \div \alpha, \tag{S1}$$

where A_{distance} and A_{voltage} are the amplitude in nm and V, respectively; S_{N} is the normal sensitivity of the photodetector, which can be measured in force spectroscopy by AFM; α is amplification factor of Bruker ICON AFM system, which is set as 16 to achieve higher sensitivity for the whole experiments. If we obtained an amplitude as 25 mV with the sensitivity S_{N} measured of 206 nm/V, it is convenient to transform into the amplitude of distance 0.025 V × 206 nm/V ÷ 16 = 0.322 nm.

For force calibration, we use the formulas from the book "B. Bhushan, Nanotribology and Nanomechanics, 2008"[1]. The normal force F_{N} and lateral force F_{L} are calibrated as below:

$$F_N = \frac{Ewd^3}{4l^3} \cdot S_{\mathrm{N}} \cdot V_N, \tag{S2}$$

$$F_L = k_{\mathrm{L}} \cdot S_{\mathrm{L}} \cdot V_L, \tag{S3}$$

where E is the elastic modulus of the cantilever, here we use the values of silicon for $E = 1.69 \times 10^{11}$ N/m^2 [1]; l, w and d are the length, width and thickness of the cantilever, here we use the values of l = 450 μm, w = 50 μm, d = 2 μm, respective; h is the tip height, here we use the value of h = 11.5 μm measured from SEM image of Fig. S2a; V_{N} and V_{L} are the normal and lateral deflection recorded by the quadrant photodetector; k_{L} and S_{L} are the lateral stiffness and sensitivity of the photodetector, respectively, which can be obtained by the InvOLS calibration[2,3]:

$$k_{\mathrm{L}} = \frac{k_\theta}{(h + \frac{d}{2})^2}, \tag{S4}$$

$$S_L = (h + \frac{d}{2})\sqrt{\frac{2k_{\mathrm{B}}Tk_\theta}{\pi\omega_{\mathrm{t}}N_{\mathrm{white}}^2 Q}}, \tag{S5}$$

where k_θ is the torsional torque constant in radian, which can be obtained from Sader's online calibration website (http://www.ampc.ms.unimelb.edu.au/afm/calibration.html); k_{B} is Boltzmann constant, which is 1.3806 × 10^{-23} J/K; T is temperature, which is set as 300 K (room temperature); ω_{t} is the torsional frequency, here measured as 197.634 kHz; Q is the quality factor, here measured as 293.8; N_{white} is the white noise, here measured as 3.93 × 10^{-8} V/$\sqrt{\mathrm{Hz}}$. The detailed calculations of quality factor and white noise of the cantilever can be referred to the previous literature.[4–6] Then, the friction force can be calculated as the half of F_{L} (trace) minus F_L (retrace), *i.e.* the area enclosed by the hysteresis loop of the lateral force is equal to the work done by friction force. The frictional hysteresis loops we obtained in C-EFM seem no big difference from the static contact mode. Thus, we performed the same force calibration for static and dynamic measurements.

2. The graphene failure after higher AC bias scanning

As we mentioned in the manuscript, only with AC bias will be harmful for the sample surface. That is why we try to add DC bias here to avoid this damage, which is not even solved in the previous studies[7,8]. The failure process of 3L graphene surface by wear is measured by C-EFM shown in Fig. S1, with the AC bias ranging from 0 to 6 V and the fixed V_{DC} = 0 V, ω = 24 kHz and F_{N} = 10 nN. Although the trilayer graphene is not our main discussion target, it is quite essential to verify the importance of introducing DC bias. Obviously, for V_{AC} = 6 V, the scanning becomes very unstable and we observe clear wear of graphene through optical microscope afterwards (see Fig. S1(i)). So we believe the resistance against AC bias of monolayer graphene should be lower, as announced of "higher AC bias over 3 V will lead to the high friction and subsequent wear"[9].

3. Contact resonant frequency sweep of the cantilever

Fig. S2a shows the side view of the tip from the scanning electron microscope (SEM), with the zoom-in tip apex shown in Fig. S2b. The tip radius is measured roughly to be ∼15 nm. The contact resonant frequency sweep of the softer cantilever (Bruker, SCM-PIC, the spring constant $k \approx 0.2$ N/m, the free resonant frequency $\omega_0 \approx$ 10 kHz) is the next compulsory process when the tip approaches the surface by C-EFM. It can be carried out by frequency sweep if setting with an appropriate center value and range. As shown in Fig. S2c, the amplitude

displays a peak at AC frequency of 31.968 kHz as well as the phase change with 180°. The 3∼5 times enlarged resonant frequency from free to forced vibrations is according well with theoretical predictions [9]. For simplicity, the resonant frequency $\omega_{\rm res}$ is set as 32 kHz in the whole work with the maximum frequency range of $\omega_{\rm res}$ ± 16 kHz.

Generally, there is no evidence or references to show the contact resonant frequency is dependent on DC bias. Thus, when$V_{\rm DC}$ is not discussed, *e.g.* the applied DC bias for Fig. 2e, it is always set as 0 V.

Considering the contact resonant frequency $\omega_{\rm res}$ of 32 kHz, we set ω as 30kHz in Fig. 2c. Actually, we carried both experiments at 30 kHz and 32 kHz, but find the friction values decreased uniformly at $V_{\rm DC}$ = 7 V for 50% ($V_{\rm AC}$ = 2 V) and 75% ($V_{\rm AC}$ = 4 V). As shown in the error bars Fig. 2c, it does not show too much influence on the measurements at ω = 30 kHz. On the other hand, even at 30 kHz, we can see that the amplitude jumps to over 1.5 nm for $V_{\rm DC}$ ≈ -5 and 7 V. The AFM engineers also suggest not to measure over 2 nm amplitude in the contact mode. Therefore, considering the instrument's issues and the validity of the results, we decided to apply 30 kHz for Fig. 2c.

As observed in Fig. 2e, we can see that friction and amplitude are influenced within the ω range from 28 to 36 kHz, *i.e.* $\omega_{\rm res}$ ± 4 kHz. Out of this range, both parameters are exempted from altering ω. Thus, 2ω ranging from 56 to 72 kHz is far from our measurements and can be ignored in our work.

4. Work function of monolayer graphene measured by KPFM

To confirm the contact potential difference between the Pt/Ir coated silicon tip and the monolayer graphene surface, KPFM measurement was carried out via a harder cantilever (Bruker, SCM-PIT, the spring constant k ≈ 2.8 N/m, the free resonance frequency ω_0 ≈ 75 kHz) to obtain enough excitation signals. Fig. S3a and b show the topography and bias image of the monolayer graphene stripe on SiO_2/Si, respectively, with the line profiles of the height and bias shown in Fig. S3c. Obviously, the contact potential of the Pt/Ir coated tip is higher than the graphene surface with the value of ∼1.06 V. It fits well with the work functions ϕ of Ir(111) and monolayer graphene with the difference of ∼1.2 eV [10]. Thus, the contact potential difference can be calculated as $V_{\rm CPD} = V_{\rm tip} - V_{\rm Gr} = (\phi_{\rm tip} - \phi_{\rm Gr})/{\rm e}$ = 1.06 V [11], leading to the system compensated potential $V_{\rm DC}$ as positive value (∼1.1 V) to eliminate the amplitude. However, in KPFM, the measured potential difference of negative value (-1.06 V) is due to the bias compensation on the tip to equal with the graphene surface, *i.e.* $V_{\rm tip} - 1.06\ {\rm V} = V_{\rm Gr}$.

As known, the compensated potential difference (CPD) measured by KPFM reflects the work function distinction between tip and graphene, and may change for various reasons. For preparation conditions, graphene synthesized on different substrates during CVD growth (*e.g.* Cu, Ni or SiC) can exhibit substrate-induced doping, which affects the CPD; Next, mechanical or chemical transfer of graphene can introduce residues (*e.g.* polymers, water) or create wrinkles. These extrinsic factors can modify the local electronic environment, causing CPD heterogeneity. In addition, thermal or plasma cleaning can reduce adsorbates and residues, leading to a more uniform CPD. Conversely, poor cleaning can retain contaminants that also affects CPD values.

5. Normal force tuning on friction via electric coupling

The resonant friction and amplitude tuned by the normal force from 10 nN to 30 nN are shown in Fig. S4a and b with the AC bias of 2 V and the AC frequency range of $\omega_{\rm res}$ ± 16 kHz, respectively. Besides the friction drop Δf at $\omega_{\rm res}$ decreases with higher loading shown in Fig. 2a, the friction force at the non-resonant area away from $\omega_{\rm res}$ ± 4 kHz increases linearly with the normal force as usual. The corresponding amplitude at the non-resonant area maintains below 0.1 nm, which is difficult to affect the friction force. In addition, the tuned resonant friction and amplitude by the normal force plus the AC bias are shown in Fig. S4c and d, respectively, with the AC frequency at $\omega_{\rm res}$ − 2 kHz (30 kHz) and the AC bias range of 0∼3 V. The friction force decreases linearly with AC bias, with less steeper slopes for the higher normal loading. While the amplitude increases with AC bias, but still fits negatively correlated with the resonant friction under the varying normal force.

6. The selection of fitted simulation parameters

The generalized Prandtl-Tomlinson (PT) model is selected as the simulation method in our work, rather than the thermally activated PT model. Two issues led us to adopt the approach described in this paper: (1) a conventional one-dimensional model fails to accurately capture the phenomena observed in our experiments [12,13]; (2) introducing vibrational effects in terms of energy into the simulation yields results that do not align with the experimental data [14,15].

The parameters in our model can be divided into two categories: one describes how the electric field modulates vibration, and the other adjusts the generalized PT model after the vibration is introduced. As displayed in Eq. (4),

the vibration amplitude is determined by three parameters: α, ω_{res} and ζ. The parameter ω_{res} is directly set as 32 kHz based on experimental observations, while α and ζ are obtained through fitting the vibration datasets. The reference datasets include two groups: the frequency-amplitude dataset A_ω measured under conditions of $V_{\mathrm{AC}} = 2$ V, $V_{\mathrm{DC}} = 0$ V and the DC voltage-amplitude dataset V_{v} measured at $\omega = 32$ kHz, $V_{\mathrm{AC}} = 2$ V. The experimental results are denoted as A_ω^{exp} $A_{\mathrm{v}}^{\mathrm{exp}}$, respectively. Then, the objective function is defined as Eq. (S6):

$$\Phi_{\mathrm{A}} = ||A_\omega(\alpha, \zeta) - A_\omega^{\mathrm{exp}}||_2 + ||A_{\mathrm{v}}(\alpha, \zeta) - A_{\mathrm{v}}^{\mathrm{exp}}||_2, \tag{S6}$$

where $||\cdot||_2$ represents the Euclidean norm, used to quantify the differences between the model and experimental data. The parameters α and ζ are determined by minimizing Φ_{A} using the interior-point algorithm implemented in MATLAB[16,17]. The fitting results are validated by comparing the model-predicted frequency-amplitude data for $V_{\mathrm{AC}} = 3$ V, $V_{\mathrm{DC}} = 0$ V and the DC voltage-amplitude data for $\omega = 32$ kHz, $V_{\mathrm{AC}} = 4$ V with the corresponding experimental data, while the absolute errors are less than 0.05 nm.

The remaining parameters, which are directly obtained from experimental calibration, include the slip velocity v, the normal and lateral spring stiffness constants k_{x} and k_{z} as well as the graphene lattice constant a. The mass m, lateral and normal dissipation μ_{x} and μ_{z}, and the parameters $\sigma \in 0$ for calibrating the interface potential energy were all fitted using a method similar to the one described above.

After directly incorporating the linear vibration mode into the one-dimensional PT model in the form of energy, the simulation results are shown in Fig. S5. However, this result does not fully align with our expectations, as we anticipated a drop near a certain resonance frequency. The basic form of the one-dimensional PT model is given by Eq. (S7):

$$m\frac{d^2x}{dt^2} + m\mu\frac{dx}{dt} + \frac{dE}{dx} = \xi(t). \tag{S7}$$

The effect of vibration in the simulation was regarded as a perturbation to the potential energy, *e.g.* $E = E_0(1+\alpha\cos 2\pi\omega t)$, which oscillated with a frequency ω and had a relative modulation factor α. Both the calculations in our study and the previous findings[9,18,19] indicate that this approximation struggles to capture the friction reduction caused by frequency, rather than the modulation factor α, within the frequency range considered in this work.

References

[1] B. Bhushan, *Nanotribology and nanomechanics: an introduction*, Springer **2008**.
[2] N. Mullin, J. K. Hobbs, *Rev. Sci. Instrum.* **2014**, *85*.
[3] C. P. Green, H. Lioe, J. P. Cleveland, R. Proksch, P. Mulvaney, J. E. Sader, *Rev. Sci. Instrum.* **2004**, *75*, 1988.
[4] K. Y. Yasumura, T. D. Stowe, E. M. Chow, T. Pfafman, T. W. Kenny, B. C. Stipe, D. Rugar, *J. Microelectromech. S.* **2000**, *9*, 117.
[5] S. Rast, C. Wattinger, U. Gysin, E. Meyer, *Nanotechnology* **2000**, *11*, 169.
[6] J. E. Sader, J. Sanelli, B. D. Hughes, J. P. Monty, E. J. Bieske, *Rev. Sci. Instrum.* **2011**, *82*.
[7] M. A. Lantz, D. Wiesmann, B. Gotsmann, *Nat. Nanotechnol.* **2009**, *4*, 586.
[8] E. Gnecco, A. Socoliuc, S. Maier, J. Gessler, T. Glatzel, A. Baratoff, E. Meyer, *Nanotechnology* **2008**, *20*, 025501.
[9] A. Socoliuc, E. Gnecco, S. Maier, O. Pfeiffer, A. Baratoff, R. Bennewitz, E. Meyer, *Science* **2006**, *313*, 207.
[10] Z. Liu, A. Hinaut, S. Peeters, S. Scherb, E. Meyer, M. C. Righi, T. Glatzel, *Nanomaterials* **2022**, *12*, 968.
[11] S. Choi, Z. Shaolin, W. Yang, *J. Korean Phys. Soc.* **2014**, *64*, 1550.
[12] Y. Dong, A. Vadakkepatt, A. Martini, *Tribol. Lett.* **2011**, *44*, 367.
[13] A. Vanossi, N. Manini, M. Urbakh, S. Zapperi, E. Tosatti, *Rev. Mod. Phys.* **2013**, *85*, 529.
[14] J. Cao, Q. Li, *Friction* **2022**, *10*, 1650.
[15] X. Ma, X. Tan, D. Guo, S. Wen, *Friction* **2023**, *11*, 1225.
[16] R. H. Byrd, J. C. Gilbert, J. Nocedal, *Math. Program.* **2000**, *89*, 149.
[17] R. A. Waltz, J. L. Morales, J. Nocedal, D. Orban, *Math. Program.* **2006**, *107*, 391.
[18] O. Fajardo, E. Gnecco, J. Mazo, *Phys. Rev. B* **2014**, *89*, 075423.
[19] H. Iizuka, J. Nakamura, A. Natori, *Phys. Rev. B* **2009**, *80*, 155449.

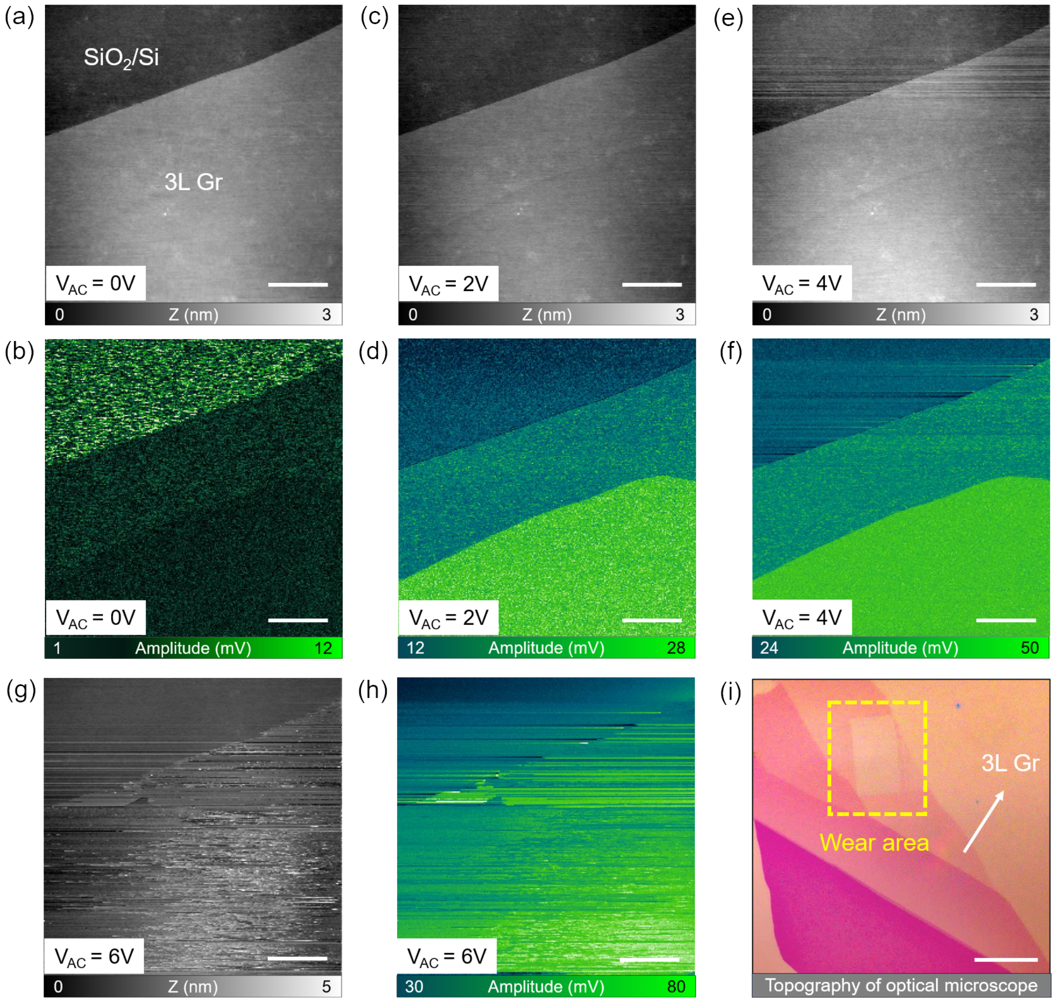

Figure S1. Topography and amplitude of 3L graphene measured by C-EFM (F_N = 10 nN, ω = 24 kHz, V_{DC} = 0 V) under: (a) and (b) V_{AC} = 0 V. (c) and (d) V_{AC} = 2 V. (e) and (f) V_{AC} = 4 V. (g) and (h) V_{AC} = 6 V. Scale bars: 4 μm.

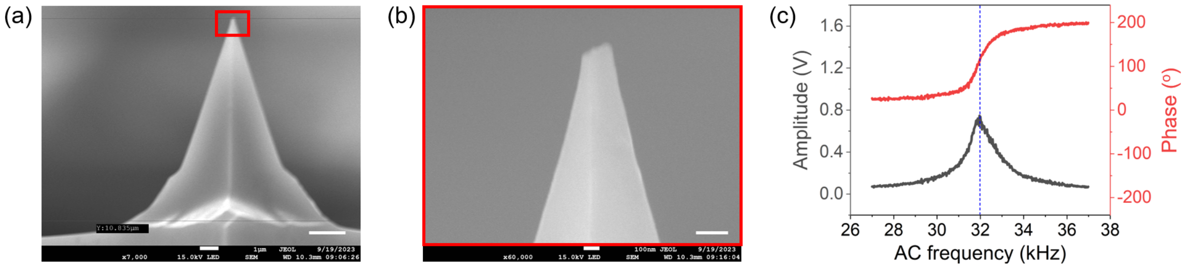

Figure S2. Tip and cantilever information. (a) SEM image of tip in side view, with the zoom-in details shown in (b). (c) Resonant sweep of cantilever by C-EFM to search the resonant frequency peak. Scale bars for **a**: 2 μm and **b**: 200 nm.

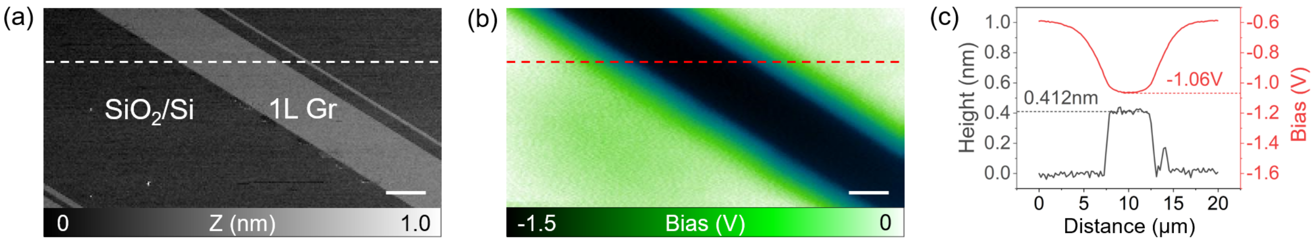

Figure S3. KPFM measurement of monolayer graphene flake on SiO_2/Si substrate. (a) Topography. (b) Contact potential. (c) Height and bias profiles from (a) and (b). Scale bar: 2 μm.

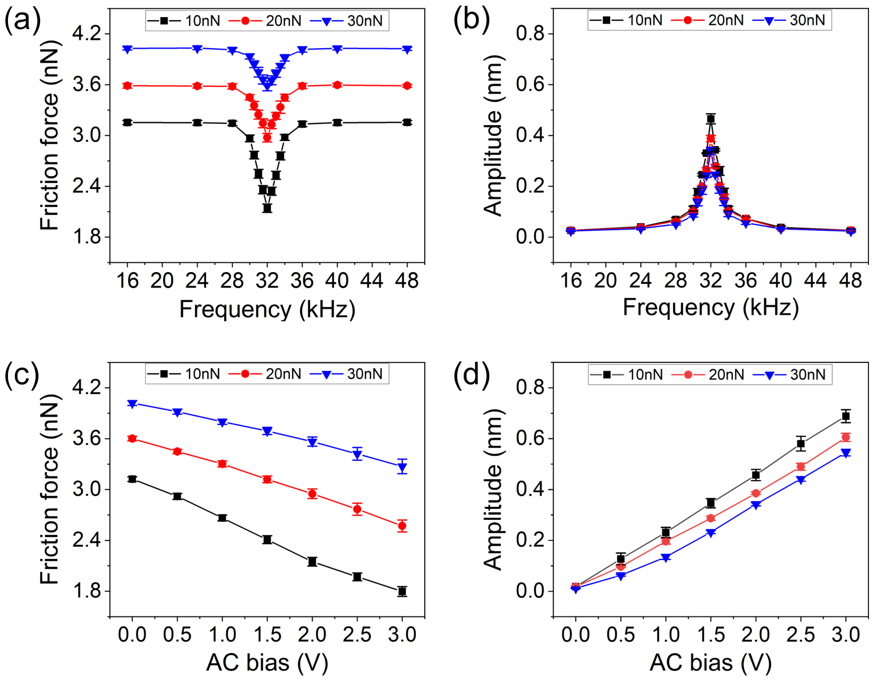

Figure S4. Friction force and amplitude of monolayer graphene tuned by: (a) and (b) AC frequency with the normal force at 10 nN, 20 nN and 30 nN, respectively. (c) and (d) AC bias with the normal force at 10 nN, 20 nN and 30 nN, respectively.

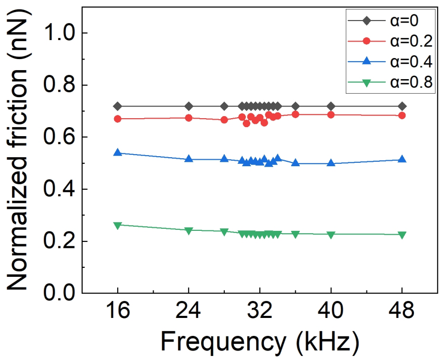

Figure S5. Numerical simulation using the PT friction model with the different modulation factor α.
